# Projecting the COPD population and costs in England and Scotland: 2011 to 2030

**DOI:** 10.1038/srep31893

**Published:** 2016-09-01

**Authors:** Susannah McLean, Martine Hoogendoorn, Rudolf T. Hoogenveen, Talitha L. Feenstra, Sarah Wild, Colin R. Simpson, Maureen Rutten-van Mölken, Aziz Sheikh

**Affiliations:** 1 Allergy and Respiratory Research Group, Centre for Population Health Sciences, University of Edinburgh, Doorway 1, Medical Quad, Teviot Place, Edinburgh, EH8 9AG, Scotland, UK; 2 Institute for Medical Technology Assessment, Erasmus University, Rotterdam, The Netherlands; 3 National Institute for Public Health and the Environment, Bilthoven, The Netherlands

## Abstract

We aimed to estimate the prevalence, healthcare costs and number of deaths among people with chronic obstructive pulmonary disease (COPD) in England and Scotland 2011–2030. We adapted the Dutch COPD Model by using English and Scottish demographic, COPD incidence, COPD prevalence, smoking prevalence and mortality data to make projections. In England, the prevalence of COPD was estimated to be 1.79% (95% uncertainty interval 1.77–1.81) in 2011, increasing to 2.19% (1.85–2.33) by 2030. In Scotland, prevalence was 2.03% (1.96–2.10) in 2011 increasing to 2.20% (1.98–2.40) in 2030. These increases were driven by more women developing COPD. Annual direct healthcare costs of COPD in England were estimated to increase from £1.50 billon (1.18–2.50) in 2011 to £2.32 (1.85–3.08) billion in 2030. In Scotland, costs increased from £159 million (128–268) in 2011 to £207 (165–274) million in 2030. The deaths in England were estimated to increase from 99,200 (92,500–128,500) in 2011, to 129,400 (126,400–133,400) by 2030. In Scotland, in 2011 there were 9,700 (9,000–12,300) deaths and 13,900 (13,400–14,500) deaths in 2030. The number of people with COPD will increase substantially over the coming years in England and Scotland, particularly in females. Services need to adapt to this increasing demand.

Chronic obstructive pulmonary disease (COPD) poses a substantial healthcare burden on many countries. The most recent figures from the Global Burden of Disease Study 2010^1^ show that it is now the third leading global cause of death^2^.

Projecting the future numbers of people who will suffer chronic disease is essential for governments to plan their healthcare budgets and resource deployment.

In order to inform modelling for England and Scotland, we undertook a systematic review to identify and evaluate models that estimate the prevalence and COPD burden^3^,^4^. We identified 22 such models which used a range of techniques. Three related models scored highly for quality; they were developed by Erasmus University, Rotterdam, and RIVM, Bilthoven, the Netherlands, and cross validated with other models^5^,^6^,^7^,^8^. Following contact with the authors we agreed that we could use their most up-to-date “Dutch COPD Model” with English and Scottish data to produce projections for the prevalence, costs and number of deaths from COPD over the period 2011–2030.

## Methods

### The Dutch COPD Model general description

**Figure 1:**
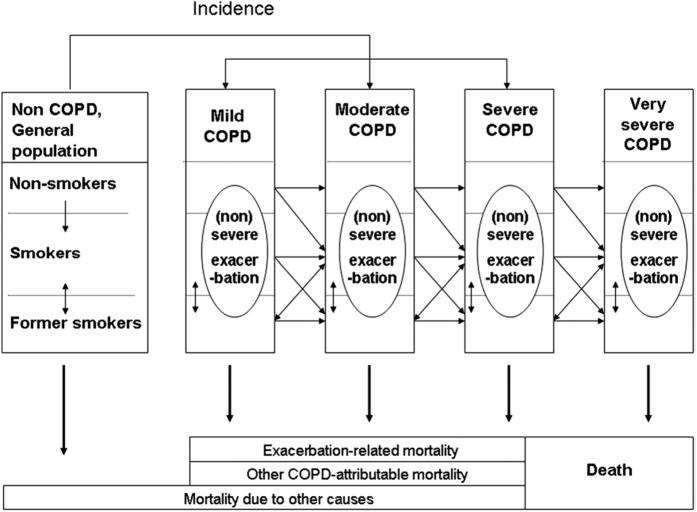
The Dutch COPD Model (reproduced with permission from Hoogendoorn, M., Rutten-Van Molken, M. P. M. H., Hoogenveen, R., Maiwenn, J. & Feenstra, T. L. Developing and applying a stochastic dynamic population model for chronic obstructive pulmonary disease. *Value Health.* 14, 1039–1047. Epub 2011 Sep 1022). (2011).

The Dutch COPD Model was developed by The Institute for Medical Technology Assessment, Erasmus University, Rotterdam, The Netherlands and The National Institute for Public Health and the Environment, Bilthoven, The Netherlands. It has been described in detail in several publications^6^,^7^,^9^,^10^. Figure 1 shows the structure of the Dutch COPD Model. Figure 1 should be read from left to right and top to bottom. The model is a multi-state model describing the following states: no COPD, mild, moderate, severe, very severe and death. The model follows COPD patients over the course of disease from incidence until death. In the model incidence, prevalence, mortality, progression and health care costs of COPD are specified by 2007 Global initiative for Obstructive Lung Disease (GOLD) severity stage^11^.

The model follows a cohort starting out with no COPD but with specific age, sex and smoking rates, then, year on year, members of each cohort develop COPD. For each cohort the prevalence and incidence of COPD, within this population, is calculated. Each year a new birth cohort is added to the non-COPD population and existing cohorts age one year. The annual incidence of new cases of COPD is modelled taking into account the start, stop and restart rates of smoking within the general population. Prevalent COPD cases are modelled to progress to worse severity stages over time and changes in smoking status can occur. Each COPD severity state is associated with a certain risk to die of COPD or another cause. In addition, patients in each COPD severity state have a risk of experiencing exacerbations. This risk increases with increasing COPD severity. Healthcare costs are calculated as the costs for maintenance treatment specified by severity state and the costs of treating exacerbations. Main outcomes of the model are number of COPD patients, number of deaths, and COPD-related treatment costs over time. Extensive details about the model can be found in previous publications about the model and in the Supplement. The adapted input parameters are described below.

### Starting point

The starting point of the current model simulation was the English or Scottish population in the baseline year of 2011 in terms of demographics (age and sex), smoking status (smokers, former smokers and never- smokers) and the incidence and prevalence of COPD by age and sex in one year age classes.

### Use of English and Scottish input data-ethics and governance issues

English and Scottish input data were required for a variety of inputs (Table S1 in the Supplement). Data sources were identified through discussion between the teams in Edinburgh and Rotterdam. A National Health Service (NHS) Research Ethics Committee approval waiver was granted for use of the English and Scottish data, on the understanding that the data were anonymised. Permission to use Clinical Practice Research Datalink (CPRD) data was granted from the Independent Scientific Advisory Committee (ISAC) of CPRD, which oversees all applications to CPRD (Approval 10-084RA). An approval waiver for analysis required for the Lothian COPD Cohort was granted by the Privacy Advisory Committee (PAC) at Information Services Division (ISD). Caldicott Guardian permission for aggregated data from the Lothian COPD Cohort to be used outside the UK was also granted. The Supplement contains tables of input data and details of calculations based on the data.

### Demographic details for England and Scotland

Country specific demographic data were obtained from the Office for National Statistics for England (Table S2 in the Supplement) and the General Register’s Office for Scotland (Table S3).

### The incidence of COPD

Incidence of COPD under 35 years of age was negligible. In those aged over 35, the incidence of COPD in England was calculated using the CPRD dataset. CPRD is a large UK-based dataset of routinely collected health data, funded by the National Institute for Health Research (NIHR) and the Medicines and Healthcare Products Regulatory Agency (MHRA). CPRD extracts data from electronic medical records in primary care. We used the CPRD dataset that was used to develop a prognostic model for COPD^12^. With permission from CPRD, the analysis of Kotz *et al*.^12^, was extended to provide incidence and prevalence estimates for COPD in primary care (further details in the Supplement). The dataset contained 38,606 patients coded as COPD (Tables S4 and S5 in the Supplement). We used data from only the English patients in this database to calculate the incidence for England.

For Scotland, the incidence was calculated from the Lothian COPD Cohort Database. This database consists of all new diagnoses of COPD in the region of Lothian between 2000 and 2008. This incidence was adjusted by Scottish Index of Multiple Deprivation (SIMD) to apply to the whole of Scotland. (Table S6).

The incidence of COPD in each age and gender class was distributed over the three smoking classes using the number of smokers in each class and the relative risks of smokers and former smokers of having COPD. The frequency distribution of the FEV1% predicted among the incident cases was estimated by the model and defined as the distribution that, given disease progression and mortality, would not change the FEV1 predicted among the prevalent cases in the first year of the model. Therefore 40% of newly diagnosed COPD patients had mild COPD 55% had moderate, 4% had severe and 0.1% had very severe.

### The prevalence of COPD

CPRD was used to estimate the prevalence in one year age and sex groups for English patients (Table S7).

All COPD patients should be reviewed at least once a year under the terms of the Quality and Outcomes Framework (QOF) of the GP General Medical Services Contract in both England and Scotland^13^. Therefore, a reasonable proxy for the prevalence was the number of people consulting a general practitioner (GP) or nurse at least once during a 15 month period for COPD divided by the mid-point practice population.

For Scotland, this number was obtained from the Practice Team Information (PTI) Database, which comprises of a sample of approximately 6% of Scottish GPs who download their data to Information Services Division (ISD), a special health board in Scotland. The codes selected were the codes used to allocate QOF payments to GPs (Table S8). To ensure that this prevalence was accurate the SIMD was used to correct the PTI data to the whole of Scotland according to deprivation quintiles (Table S9).

The prevalence of COPD in each age and gender class is distributed over the three smoking classes using the number of smokers in each smoking class and the relative risks of smokers and former smokers to have COPD. It is assumed that the relative risks of smokers and former smokers to get COPD is equal to the relative risk of having COPD. The prevalence of COPD in each age, sex and smoking class is further distributed over the four GOLD stages. This was inputted from a frequency distribution of FEV1% predicted over all COPD classes obtained from Dutch GP data. Based on a normal distribution with a mean FEV1% predicted of 68.3% (SD 19.9%) it was estimated that 27% had mild COPD 55% had moderate COPD, 15% had severe COPD and 3% had very severe COPD. Frequency distributions of English and Scottish severity were not available at the time of the analysis however, subsequently, we have confirmed that the severity distribution in a representative English dataset was mean FEV1% predicted of 68.9% (SD 17.3%) (Health Survey for England 2010)^14^ and so very similar to the Dutch distribution. The severity distribution of the Scottish data was later confirmed as mean FEV1% predicted 59.0% (SD 18.8%) (Lothian COPD Cohort).

### Smoking

Each year transitions between smoking stages occur. Non–smoking patients could start smoking, smoking patients could stop smoking and former smoking patients had a certain probability to restart smoking.

The General Lifestyle Survey for England 2011^15^ provided estimated proportions of current and former smokers for England in adults. For under 16 s, data were retrieved from the Health Survey for England 2010 (Table S10)^14^.

The proportion of the Scottish adult population who were smokers, former smokers and never smokers was obtained from the Scottish Health Survey 2011^16^. Data for under 16 s were extracted from the Scottish Schools Adolescent Lifestyle and Substance Use Survey 2010 (Table S11)^17^.

### Additional smoking data: Start, stop and restart probabilities for smoking

Models for England and Scotland both used the following data:

- Numbers and ages of starting smoking from the Avon Longitudinal Study of Parents and Children (ALSPAC), a cohort study of over 14000 mothers, partners and children who were enrolled during their pregnancies in 1991/2. Approximately 30% of the parental male and female cohort were smokers^18^. The whole cohort had been asked whether they were smokers and at what age they had started smoking. We used this to generate an age related probability of starting smoking within the cohort. We directly transferred this probability to the populations of Scotland and England.
- For stopping smoking, we used the proportion of the total population, by age and sex, who were smokers who had successfully stopped for over a year from the Smoking Toolkit Survey (Table S12)^19^.
- Restarting smoking probabilities were used from the original Dutch Model as no equivalent survey was identified for England or Scotland. These data came from an annual Dutch Survey of smokers^20^.

### Relative risks of smokers and non-smokers

The age- and sex-specific relative risks of smokers and non-smokers to develop COPD were left the same as in the original Dutch Model. This was because the original data came from the United States Surgeon General’s report and such risks were assessed as being consistent across populations, being more to do with the biology of COPD than with epidemiology^10^,^21^,^22^.

### Disease progression

In those with established COPD there is a probability to progress to the next level of COPD severity. This disease progression was modelled as an annual decline in FEV1% predicted depending on age, sex, smoking status and FEV1% predicted. This annual decline is based on a random effects analysis of 5,000 patients in the Lung Health Study^23^. Details of the calculation of lung function decline are published in the Appendix to the 2005 Hoogendoorn *et al*. European Respiratory Journal article^7^. The values found were used in two ways: firstly, to describe the change of the FEV1 distribution within a particular COPD state, and subsequently, to calculate the transition rates between COPD states. This first use of the lung function decline values is the unique feature of our model. Exacerbations accelerate lung function decline. Disease progression was not adapted for the current study.

### Exacerbations

Each severity stage is associated with a risk for a moderate or a severe exacerbation as determined by previous work^24^. A moderate exacerbation was defined as necessitating a prescription of antibiotics and steroids. A severe exacerbation was defined as a hospital admission^10^. Exacerbations accelerate lung function decline and there was also an exacerbation related mortality which was calculated from a previous meta-analysis by the Dutch team^25^. Exacerbation probabilities in the model were based on a systematic review and meta-analysis of about 20 studies and were not adapted for the current study^24^.

### Costs

The direct healthcare costs of caring for people with COPD were divided into maintenance costs for the ongoing care of people with COPD and the additional costs for moderate or severe exacerbations. Costs associated with maintenance treatment for COPD were specified by GOLD COPD stage during the modelling. They were obtained from a micro-costing for a cost utility analysis of indacaterol, which gave list prices and an estimate of average annual usage statistics that came from a large COPD patient database or from expert opinion via a Delphi process^26^. Multiplying cost by usage generated an average total cost per severity stage (Table S13). Maintenance costs were calculated by multiplying the annual number of patients alive with the COPD-related maintenance costs specified by COPD severity stage.

Costs related to exacerbations came from a tiotropium study’s Delphi panel’s estimates for exacerbations in England and Scotland^27^. The authors calculated that the cost of a moderate exacerbation was £118 and the cost of a severe exacerbation, was £3,726 in England and £3,329 in Scotland. Exacerbation-related costs were calculated as the model projected numbers of moderate and severe exacerbations each year multiplied by the cost per exacerbation. Cost per patient and cost per exacerbation were assumed constant over time.

### Mortality

In the model all-cause mortality among COPD patients was divided into “excess mortality” and “mortality from other causes”, where “excess mortality” was defined as the difference in mortality between COPD patients and the general population which includes the increased risk of dying from other smoking-related diseases. COPD excess mortality and mortality in the general population were used to calculate the COPD-attributable mortality in the model^9^.

The modelling parameter of “excess mortality” was calculated as detailed in the Supplement. The model estimated the annual number of deaths from all causes among the COPD population.

### Making projections

A collaboration between the authors of the Dutch COPD Model and The University of Edinburgh enabled the Dutch model to be run with input data from England and Scotland from the year 2011. Projections for the number of COPD patients specified by gender, age, smoking status and COPD disease severity and the number of exacerbations were run for 20 years to 2030. The projections were an extrapolation of current observed trends in smoking behaviour and disease progression assuming they remain constant over time.

### Sensitivity analyses

A number of one-way sensitivity analyses were performed, these demonstrate the sensitivity of the model to each parameter when altered one at a time. For the first three sensitivity analyses, we explored the impact of a 10% decrease and a 10% increase in incidence, prevalence and lung function decline. In the fourth sensitivity analysis, the severity distribution of the prevalence at baseline was shifted towards milder COPD and towards more severe COPD by changing the mean FEV1% predicted in the population by 10% in each direction. The fifth sensitivity analysis used excess mortality from another European modelling study, DYNAMO-HIA^28^,^29^. Next, an increase in the smoking cessation rate to 10% at all ages was modelled to see what effect an optimal public health policy change might have.

### Additional probabilistic sensitivity analysis

Probabilistic sensitivity analysis was undertaken to estimate the effects of uncertainty around the different input parameters on the outcomes. See Supplementary information.

## Results

### Population and prevalence with COPD

#### England

**Figure 2:**
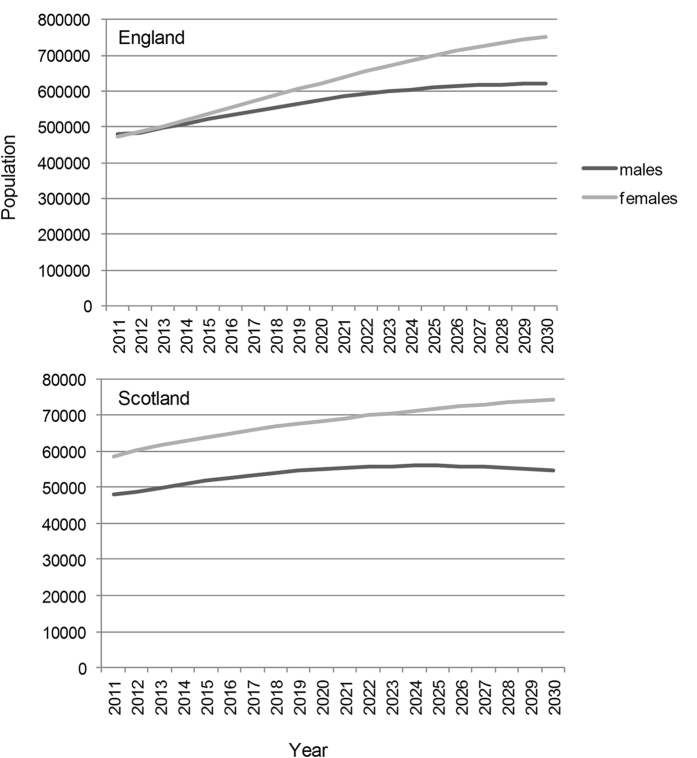
Projected number of people with COPD in England and Scotland.

For England, we estimated that the number of people with COPD would increase from 0.95 million (95% uncertainty interval 0.94–0.96 million) in 2011 to 1.3 million (1.1–1.4 million) in 2030 (an increase of 39%) (Fig. 2). The prevalence of diagnosed COPD among males was estimated as 1.8% (1.8–1.9) in 2011, increasing to 2.0% (1.7–2.1) in 2030 and 1.8% (1.7–1.8) in females in 2011 increasing to 2.4% (2.0–2.6) in females by 2030.

#### Scotland

For Scotland, we estimated that there would be 0.10 million (0.10–0.11 million) people with diagnosed COPD in 2011, increasing to 0.12 million (0.11–0.13 million) (an increase of 17%) in 2030 (see Fig. 2). The prevalence among males was estimated as 1.9% (1.8–1.9) in 2011, staying at 1.9% (1.7–2.2) in 2030 and 2.2% (2.1–2.3) in females in 2011, increasing to 2.5% (2.1–2.7) by 2030.

### Direct healthcare costs of COPD

#### England

**Figure 3:**
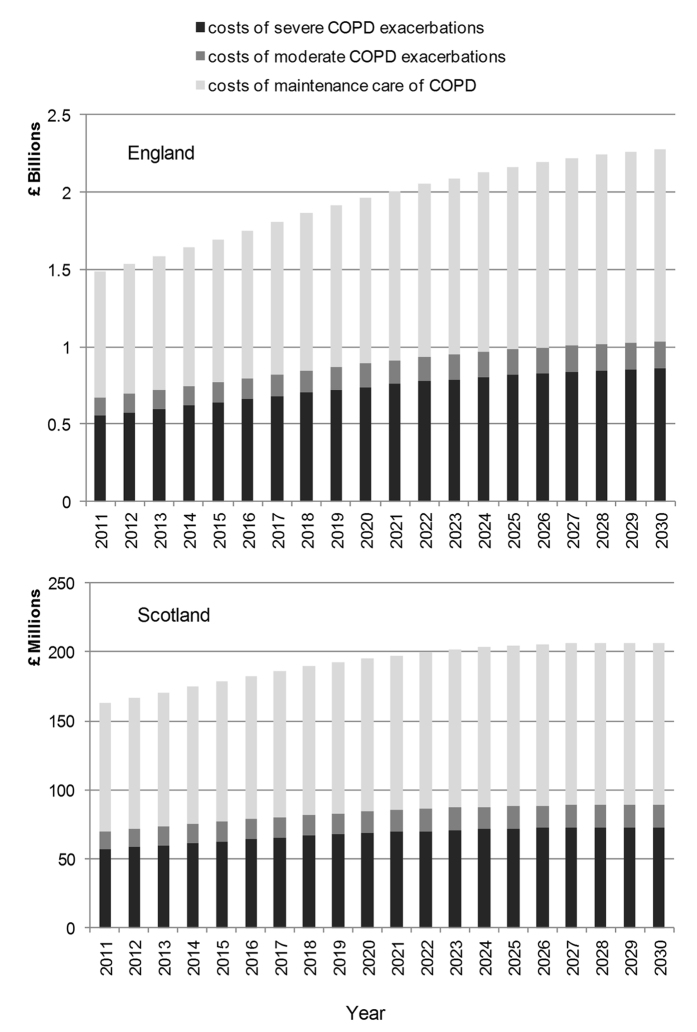
Projected direct healthcare costs of COPD.

The total annual direct healthcare costs for people with COPD including moderate and severe exacerbation costs and maintenance costs were projected to increase from £1.50 billon (1.18–2.50) in 2011 to £2.32 (1.85–3.08) billion by 2030 (Fig. 3).

#### Scotland

Total annual direct healthcare costs for caring for people with COPD were projected to increase from £159 million (128–268) in 2011 to £207 (165–274) million by 2030 (Fig. 3).

### Deaths from COPD

#### England

**Figure 4:**
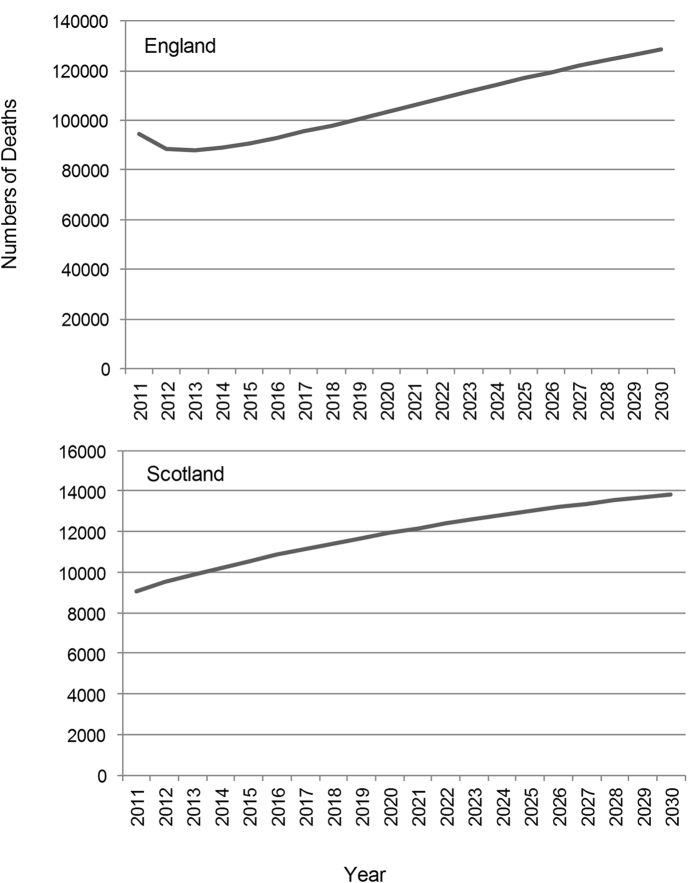
Projected number of deaths among people with COPD from all causes.

The number of deaths among people with COPD was estimated to increase from 99,200 (92,500–128,500) to 129,400 (126,400–133,400) in 2030 (Fig. 4).

#### Scotland

There were estimated to be 9,700 (9,000–12,300) deaths in 2011 and 13,900 (13,400–14,500) deaths from all causes among people with COPD in 2030 (Fig. 4).

### Sensitivity analyses

**Figure 5:**
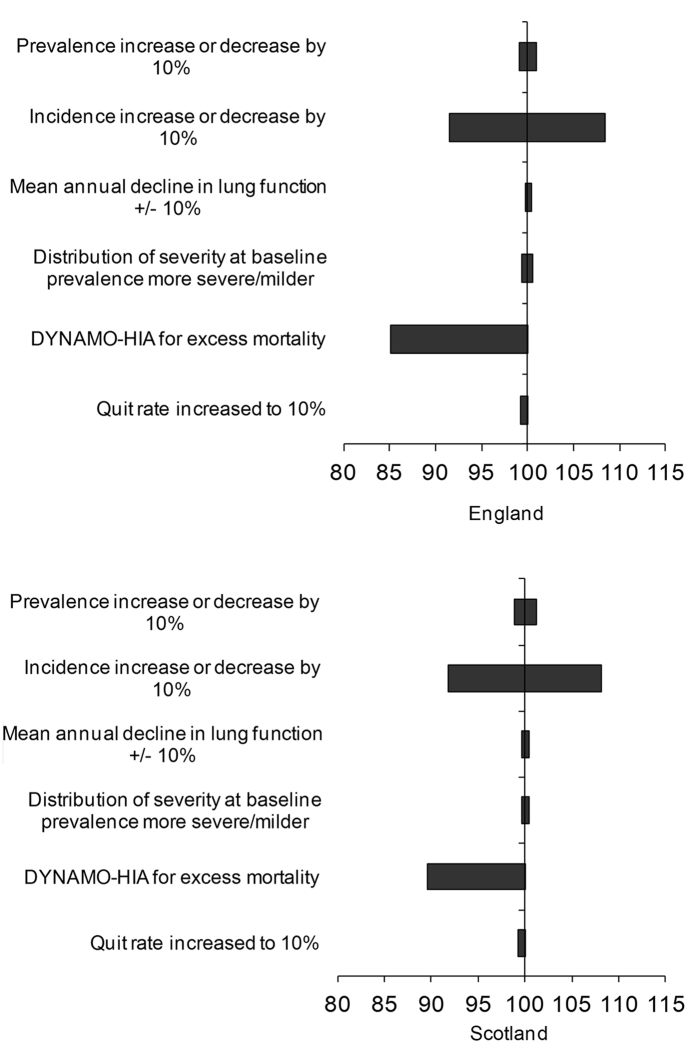
Tornado diagrams of sensitivity analysis projections of prevalence for 2030 as compared to base case (%).

The model projections were most sensitive to changes in the excess mortality of COPD. It can be seen that the sensitivity of the projection of prevalence in 2030 to one way changes in different parameters had a similar pattern for both countries from the tornado diagrams for England and Scotland (Fig. 5)^30^. It should be noted that using the DYNAMO-HIA excess mortality resulted in higher mortality rates and therefore the increase in the COPD population over time was lower compared to the base case analysis in this sensitivity analysis^29^.

### Comparison between countries

**Figure 6:**
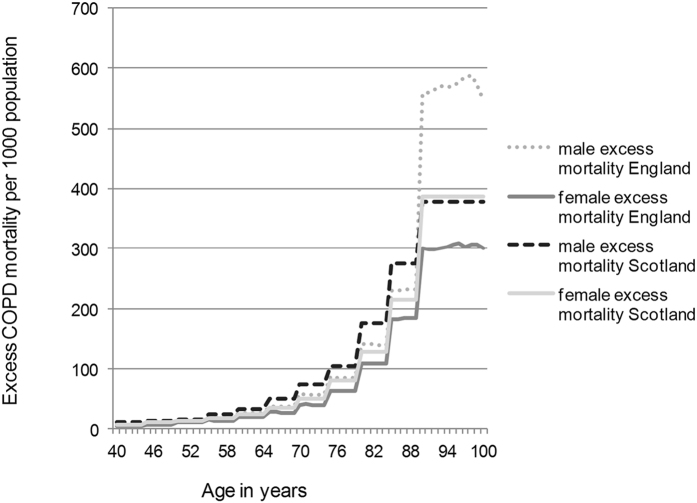
Age related excess mortality due to COPD per 1000 population.

**Figure 7:**
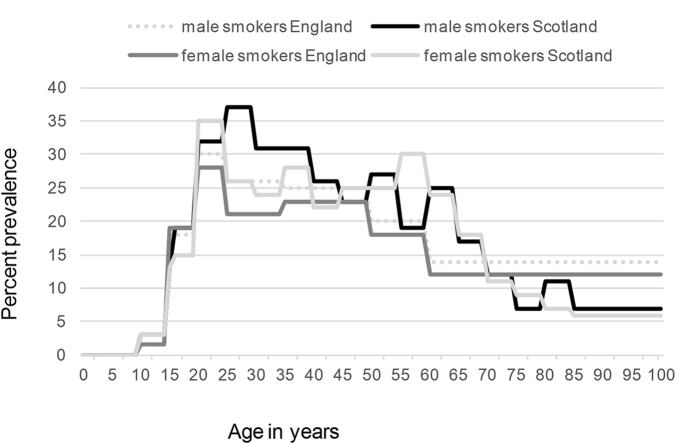
Percentage prevalence of smoking by age.

The percentage increase projected among English females with COPD is 54% between 2011 and 2030, among Scottish females this increase is 22%. Among English males there will be an estimated increase of 24%, in Scotland among males this increase is 11%. The reasons for these differences are down to differences in the modelling parameter of excess mortality calculated for each group, (Fig. 6). The excess mortality varies because English females have the longest life expectancy and the lowest rate of smoking and Scottish males have the shortest life expectancy and the highest rate of smoking, (Fig. 7). Therefore English females live longest, giving time for lung function decline to become apparent as COPD even if they are ex-smokers. Scottish males have comparatively more smokers and therefore have a high associated mortality, this means that the smokers do not live long enough to contribute greatly to the percentage increase in COPD.

## Discussion

### Principal findings

Our dynamic population models for England and Scotland projected substantial year-on-year increases in the numbers of people with COPD over the period 2011–2030. These projections are similar to the Netherlands showing an increase, especially among older female patients^7^.

As a result of the increase in the prevalence, the models projected an increase in direct healthcare costs for the overall COPD population, and also an increase in the number of deaths from all causes among people with COPD.

### Strengths and limitations

The strengths of this approach include our decision, informed by a formal systematic review, to use the Dutch COPD Model^5^,^7^ with data which had been collected for routine clinical practice; the data therefore had high validity. The Dutch COPD Model has been cross-validated with other models for hypothetical treatment scenarios^8^.

A limitation is that models structured by the GOLD severity stages in COPD have been criticised for having too narrow a view of the elements which predict COPD progression as recent arguments have been made that COPD severity is a complex mix of co-morbidity, susceptibility to exacerbations and symptoms^31^. The GOLD 2013 guidelines were updated to reflect this. Yet it has since been shown that although the 2013 GOLD classification is better than the 2007 GOLD classification at predicting exacerbations it is worse at predicting mortality and lung function decline^32^. Clinical predictive models do exist, but these are based on a few trials and so model individuals’ progression through micro-simulation^31^. These are more relevant for the evaluation of treatment options than for making projections. A further limitation is that the model did not take into account any change in diagnosis rates of COPD. Several studies have proposed that there is substantial under-diagnosis of COPD in England^33^. If this is correct, the current estimates of the COPD population are likely to be conservative. It should also be remembered that the projections are an extrapolation of currently observed trends in smoking behaviour. Stop smoking rates were obtained from the Smoking Toolkit Survey^19^ and defined as the proportion of the total population, by age and sex, who were smokers who had successfully stopped for over a year. Using this definition also means that the current projections of prevalence and costs are probably conservative estimates.

The model did not consider any changes in the cost of treatment. As a result the current projections of COPD-related healthcare costs are also likely to be conservative as in general healthcare costs increase over time.

### Implications for policy and research

In due course, it will be possible to externally validate these projections with the actual number of people with COPD. Meanwhile, these projections should be considered for planning for the increased numbers, costs and care needs of people with COPD. This will be in the context of England and Scotland facing an increasing elderly and frail population with high rates of multi-morbidity^34^.

Internationally, similar modelling using local data sources should be considered for projection of the rates and cost of COPD.

We acknowledge that policymakers also need cost-effectiveness, cost-utility and cost-benefit studies to help them allocate resources.

A general point can also be made about the challenge of representing a complex disease in the form of a state-transition Markov Model. COPD is recognised to result from the interplay of host factors, co-morbidities and the external environment. Modelling is thus an over simplification. More research and better data are needed to advance the science of epidemiological modelling.

## Conclusions

The COPD populations in England and Scotland are projected to increase substantially over the coming years to 2030. There will also be increases in the healthcare costs of COPD patients and the number of deaths among COPD patients. These increases need to be taken into account by policymakers when planning healthcare deployment and resource allocation for the future.

## Additional Information

**How to cite this article**: McLean, S. *et al*. Projecting the COPD population and costs in England and Scotland: 2011 to 2030. *Sci. Rep.* **6**, 31893; doi: 10.1038/srep31893 (2016).

## Supplementary Material

Supplementary Information
