# Supplementary material for: Projecting the COPD population and costs in England and Scotland: 2011 to 2030
Source: Sci Rep. 2016 Sep 1;6:31893. doi: 10.1038/srep31893 (PMC5008118; doi:10.1038/srep31893)
Supplement: Supplementary Information [file srep31893-s1.pdf]

Projecting the COPD population and costs in England and Scotland: 2011 to 2030

Supplementary Information

Susannah McLean[1], Martine Hoogendoorn[2], Rudolf T.Hoogenveen[3], Talitha L. Feenstra[3], Sarah Wild[1], Colin R. Simpson[1], Maureen Rutten-van Mölken[2], Aziz Sheikh[1]

1) Allergy and Respiratory Research Group, Centre for Population Health Sciences, University of Edinburgh, Doorway 1, Medical Quad, Teviot Place, Edinburgh, EH8 9AG, Scotland, UK.
2) Institute for Medical Technology Assessment, Erasmus University, Rotterdam, The Netherlands.
3) National Institute for Public Health and the Environment, Bilthoven, The Netherlands.

Corresponding Author: Susannah.mclean@ed.ac.uk

Table S1

Data inputs for Dutch Model from England and Scotland

Data Input	English Source	Scottish Source
1 year age and sex make-up of the general population in 2011	Office of National Statistics	General Register’s Office Scotland
Incidence of COPD	Clinical Practice Research Datalink	Lothian COPD Cohort
Prevalence of COPD	Clinical Practice Research Datalink	Scottish Practice Team Information Database
Smoking prevalence	The General Lifestyle Survey for England 2011 and Health Survey for England 2010	Scottish Health Survey 2011 and Scottish Schools Adolescent Lifestyle and Substance Use Survey 2010
Start smoking probabilities	Avon Longitudinal Study of Parents and Children	Avon Longitudinal Study of Parents and Children
Smoking cessation	Smoking Toolkit Survey	Smoking Toolkit Survey
Restart smoking probabilities	Original Netherlands data	Original Netherlands data
Age and sex specific relative risks of smokers and non-smokers to develop COPD	Original Netherlands data	Original Netherlands data
Modelling lung function decline	Original Netherlands data	Original Netherlands data
COPD related maintenance costs	Microcosting from UK indacaterol study	Microcosting from UK indacaterol study
COPD Excess Mortality	Calculated with relative risks from CPRD and English total mortality and prevalence	Calculated with relative risks from CPRD and Scottish total mortality and prevalence
Severity distribution of COPD	Original Netherlands data	Original Netherlands data

Demographic data

Table S2

Demographic data from the Office for National Statistics for England 2011. Only an aggregate value was available for ages 90-100 therefore this was averaged over the 10 year interval.

Age	male population	female population	male mortality	female mortality
0	347,892	331,210	0.00501	0.00375
1	342,610	327,248	0.00034	0.00030
2	338,329	323,562	0.00020	0.00016
3	342,755	326,729	0.00011	0.00010
4	332,132	316,279	0.00011	0.00010
5	325,132	310,793	0.00011	0.00007
6	311,074	297,206	0.00009	0.00008
7	305,756	291,923	0.00009	0.00005
8	297,295	282,496	0.00009	0.00005
9	291,242	277,218	0.00011	0.00006
10	297,817	285,375	0.00008	0.00004
11	307,040	291,981	0.00007	0.00008
12	316,217	301,109	0.00011	0.00009
13	320,122	305,006	0.00008	0.00007
14	329,070	313,674	0.00012	0.00009
15	331,613	312,225	0.00016	0.00009
16	330,224	313,401	0.00024	0.00011
17	342,707	324,289	0.00031	0.00017
18	343,292	328,402	0.00037	0.00017
19	353,225	344,892	0.00039	0.00019
20	367,865	352,013	0.00049	0.00018
21	365,145	348,719	0.00048	0.00023
22	361,147	353,545	0.00052	0.00023
23	365,775	364,530	0.00047	0.00024
24	355,616	361,906	0.00046	0.00023
25	366,281	363,974	0.00057	0.00026
26	369,475	367,342	0.00053	0.00027
27	361,162	362,221	0.00053	0.00035
28	364,040	368,114	0.00065	0.00028
29	364,966	369,652	0.00065	0.00030
30	369,450	372,454	0.00071	0.00036
31	371,850	373,219	0.00073	0.00040
32	358,647	357,375	0.00071	0.00045
33	335,139	332,978	0.00081	0.00047
34	330,196	327,842	0.00091	0.00051
35	334,046	334,931	0.00093	0.00057
36	340,309	341,747	0.00108	0.00056
37	345,224	348,338	0.00122	0.00062

38	360,518	363,211	0.00126	0.00070
39	375,560	378,488	0.00127	0.00074
40	385,058	393,469	0.00141	0.00083
41	376,049	383,443	0.00153	0.00099
42	384,557	393,354	0.00180	0.00103
43	384,595	392,455	0.00175	0.00117
44	391,659	396,844	0.00202	0.00120
45	389,810	399,415	0.00212	0.00134
46	392,097	401,209	0.00230	0.00154
47	388,933	397,082	0.00223	0.00148
48	381,317	388,739	0.00267	0.00172
49	373,943	379,531	0.00279	0.00191
50	361,409	366,327	0.00297	0.00209
51	346,924	351,937	0.00336	0.00235
52	339,352	344,086	0.00392	0.00252
53	332,154	336,435	0.00431	0.00270
54	319,149	324,806	0.00446	0.00314
55	308,806	313,516	0.00496	0.00320
56	297,135	303,305	0.00565	0.00365
57	297,836	305,403	0.00607	0.00404
58	295,033	301,455	0.00684	0.00411
59	285,974	295,038	0.00713	0.00494
60	288,397	297,457	0.00798	0.00534
61	293,435	305,710	0.00849	0.00559
62	302,337	313,305	0.00942	0.00608
63	319,545	332,459	0.01032	0.00665
64	348,284	361,362	0.01069	0.00713
65	268,158	280,482	0.01240	0.00815
66	260,309	273,585	0.01408	0.00896
67	259,773	273,394	0.01470	0.00964
68	240,572	256,467	0.01609	0.01055
69	213,801	231,079	0.01778	0.01168
70	191,917	210,047	0.02150	0.01369
71	199,921	219,818	0.02311	0.01435
72	197,591	219,848	0.02495	0.01655
73	192,255	214,700	0.02676	0.01711
74	181,894	206,560	0.03066	0.01987
75	171,911	198,752	0.03251	0.02195
76	162,735	191,159	0.03724	0.02475
77	149,477	180,146	0.04061	0.02837
78	140,940	174,360	0.04572	0.03206
79	136,064	172,787	0.05196	0.03545
80	127,395	168,522	0.05812	0.04083
81	116,429	160,681	0.06474	0.04548

82	104,119	148,184	0.07410	0.05288
83	92,560	136,864	0.08288	0.06032
84	83,689	127,404	0.09234	0.06755
85	74,160	118,928	0.10364	0.07565
86	63,615	108,907	0.11375	0.08718
87	54,463	99,659	0.12990	0.09761
88	46,119	90,656	0.14580	0.11009
89	39,749	82,648	0.16403	0.12414
90	10,164	27,510	0.31521	0.16014
91	10,164	27,510	0.31521	0.16014
92	10,164	27,510	0.31521	0.16014
93	10,164	27,510	0.31521	0.16014
94	10,164	27,510	0.31521	0.16014
95	10,164	27,510	0.31521	0.16014
96	10,164	27,510	0.31521	0.16014
97	10,164	27,510	0.31521	0.16014
98	10,164	27,510	0.31521	0.16014
99	10,164	27,510	0.31521	0.16014
100	10,164	27,510	0.31521	0.16014

Table S3

Demographic data from the General Register's Office for Scotland 2011. As with the English data an aggregated value for ages 90-100 was available and so this was averaged out across the 10 years.

Age	male population	female population	male mortality	female mortality
0	30,309	28,867	0.00459	0.00343
1	30,326	29,500	0.00040	0.00031
2	30,872	29,436	0.00013	0.00010
3	30,686	29,590	0.00007	0.00010
4	29,782	28,373	0.00003	0.00011
5	28,845	27,601	0.00014	0.00007
6	28,786	27,084	0.00021	0.00007
7	28,345	26,758	0.00011	0.00000
8	27,239	26,219	0.00007	0.00019
9	26,617	25,880	0.00008	0.00004
10	27,180	26,452	0.00004	0.00011
11	28,089	26,202	0.00018	0.00011
12	29,331	27,735	0.00010	0.00004
13	29,591	28,441	0.00010	0.00007
14	30,830	28,925	0.00006	0.00010
15	30,498	28,928	0.00013	0.00007
16	31,005	29,432	0.00016	0.00020
17	32,058	30,597	0.00047	0.00029
18	33,567	32,265	0.00057	0.00031
19	35,332	34,198	0.00099	0.00047
20	36,788	35,597	0.00073	0.00025
21	36,318	35,453	0.00088	0.00037
22	36,986	36,130	0.00097	0.00014
23	38,501	36,856	0.00075	0.00041
24	37,880	36,629	0.00077	0.00027
25	38,119	36,394	0.00110	0.00036
26	38,239	36,764	0.00073	0.00052
27	35,950	34,583	0.00092	0.00026
28	35,085	34,166	0.00108	0.00056
29	34,928	34,205	0.00135	0.00085
30	36,049	34,448	0.00161	0.00052
31	35,218	33,971	0.00133	0.00065
32	33,101	31,832	0.00142	0.00035
33	29,698	30,035	0.00168	0.00080
34	28,862	28,886	0.00170	0.00100
35	29,959	31,098	0.00260	0.00080
36	29,745	31,706	0.00215	0.00098
37	30,219	32,117	0.00189	0.00087
38	31,994	34,312	0.00184	0.00122
39	33,519	37,042	0.00227	0.00167

40	35,717	38,883	0.00283	0.00105
41	35,523	38,753	0.00273	0.00147
42	36,897	40,099	0.00276	0.00152
43	37,366	41,244	0.00292	0.00116
44	38,699	41,462	0.00269	0.00150
45	37,840	41,457	0.00338	0.00198
46	39,052	42,555	0.00279	0.00221
47	38,830	42,711	0.00319	0.00225
48	38,339	42,333	0.00370	0.00236
49	38,517	40,918	0.00343	0.00240
50	37,587	40,479	0.00410	0.00299
51	36,875	39,288	0.00442	0.00333
52	36,188	38,928	0.00533	0.00316
53	35,319	38,037	0.00476	0.00463
54	34,535	36,795	0.00498	0.00380
55	33,741	35,381	0.00744	0.00430
56	32,387	34,204	0.00692	0.00479
57	32,080	33,704	0.00776	0.00546
58	31,467	32,981	0.00801	0.00509
59	30,326	31,776	0.00923	0.00604
60	30,677	32,435	0.00981	0.00694
61	31,055	32,553	0.01143	0.00725
62	31,879	33,540	0.01195	0.00748
63	32,644	34,493	0.01302	0.00913
64	35,218	37,493	0.01400	0.00885
65	26,920	28,518	0.01597	0.01013
66	25,327	27,608	0.01757	0.01166
67	25,661	27,953	0.02023	0.01381
68	24,337	27,555	0.02087	0.01328
69	22,199	25,455	0.02514	0.01587
70	20,406	24,001	0.02911	0.01708
71	20,808	24,640	0.02965	0.01847
72	20,163	23,956	0.03253	0.02033
73	19,580	23,382	0.03483	0.02352
74	18,251	22,593	0.04066	0.02651
75	17,538	22,209	0.04122	0.02873
76	16,402	21,345	0.04707	0.03162
77	15,040	20,035	0.05000	0.03639
78	13,909	19,170	0.05507	0.03928
79	13,342	19,009	0.06026	0.04356
80	12,268	17,873	0.07189	0.04829
81	10,804	16,746	0.07988	0.05739
82	9,802	15,390	0.09008	0.06257
83	8,255	13,616	0.10369	0.07197

84	7,410	12,681	0.11012	0.07633
85	6,724	12,108	0.12240	0.08812
86	5,718	10,627	0.13012	0.09928
87	4,889	9,690	0.14870	0.10485
88	3,730	8,220	0.16434	0.13041
89	3,271	7,455	0.18526	0.13924
90	931	2,500	0.20797	0.20359
91	931	2,500	0.20797	0.20359
92	931	2,500	0.20797	0.20359
93	931	2,500	0.20797	0.20359
94	931	2,500	0.20797	0.20359
95	931	2,500	0.20797	0.20359
96	931	2,500	0.20797	0.20359
97	931	2,500	0.20797	0.20359
98	931	2,500	0.20797	0.20359
99	931	2,500	0.20797	0.20359
100	931	2,500	0.20797	0.20359

Incidence of COPD in CPRD database for English patients:

The study cohort was drawn from all English practices that were participating in the linkage programme when the data was extracted in 2011 that met the quality control standards.

The study population consisted of all males and females registered in eligible practices on and for at least 18 months prior to 01/01/2011 who met the quality control standard and were aged at least 35 in 2011 and had not been diagnosed with COPD.

Numerator= The number of patients with a first mention of COPD in 2011.

Denominator = Denominator data consisted of the sum of the person years of up-to-standard follow-up in 2011 of all at risk patients in the study population. The start of the observation was the 01/01/2011. The end of the observation was the minimum of the practice last collection data, the patient transfer out data, the CPRD derived death date, the first record of COPD (index date) or 31 December 2011.

The incidence rate of COPD was calculated by dividing the number of incident cases by total person time between 01/01/2011 and 31/12/2011 in the denominator population. Incidence is presented per 100,000 patient years with 95% CI calculated using the Poisson distribution and stratified by gender and age.

Table S4

Diagnosis of COPD in the CPRD dataset was based on one or more of the following Read Codes (case definition), these are high level codes and were approved by CPRD.

Read Code	Case definition
H3	COPD
H31 and below	Chronic bronchitis
H32 and below	Emphysema
H36	Mild COPD
H37	Moderate COPD
H38	Severe COPD
H3y and below	Other specified COPD
H3z	COPD not otherwise specified

Table S5

Incidence of COPD in England from Clinical Practice Research Datalink 2011. In some of the older age groups where the denominator was very small there were no incident cases of that age. This was dealt with by a smoothing process for calculating the uncertainty intervals.

Incidence rate per 100,000 person years (95% CI)		
Age	Males	Female
35	33.2 (9.1 , 85.1)	32.9 (9.0 , 84.3)
36	15.9 (1.9 , 57.5)	15.9 (1.9 , 57.5)
37	30.0 (8.2 , 76.9)	30.5 (8.3 , 78.0)
38	50.3 (20.2 , 103.6)	43.3 (15.9 , 94.3)
39	40.3 (14.8 , 87.7)	68.0 (32.6 , 125.1)
40	38.3 (14.1 , 83.4)	70.4 (35.1 , 126.0)
41	51.3 (22.1 , 101.1)	97.4 (54.5 , 160.7)
42	75.5 (39.0 , 131.9)	70.7 (35.3 , 126.5)
43	86.6 (47.3 , 145.3)	126.0 (77.0 , 194.6)
44	85.6 (46.8 , 143.7)	73.8 (38.1 , 128.9)
45	67.0 (33.5 , 120.0)	114.4 (68.9 , 178.6)
46	124.4 (77.0 , 190.2)	175.1 (117.2 , 251.4)
47	146.9 (95.1 , 216.8)	167.5 (111.3 , 242.1)
48	108.9 (64.5 , 172.1)	141.0 (89.4 , 211.6)
49	129.2 (80.0 , 197.5)	208.8 (143.7 , 293.2)
50	203.4 (139.1 , 287.1)	253.6 (180.3 , 346.6)
51	214.3 (147.5 , 301.0)	270.1 (193.8 , 366.4)
52	256.9 (181.8 , 352.6)	269.5 (191.7 , 368.4)
53	259.8 (183.9 , 356.7)	236.6 (163.9 , 330.6)
54	280.9 (200.7 , 382.5)	322.1 (234.9 , 430.9)
55	308.2 (221.1 , 418.1)	422.8 (320.2 , 547.8)
56	268.7 (187.2 , 373.7)	439.1 (331.7 , 570.2)
57	333.2 (241.2 , 448.8)	441.9 (334.7 , 572.5)
58	396.7 (295.4 , 521.6)	396.4 (295.2 , 521.3)
59	480.9 (367.9 , 617.7)	500.2 (384.3 , 639.9)
60	563.5 (438.5 , 713.2)	479.8 (365.3 , 619.0)
61	562.5 (437.7 , 711.9)	443.3 (334.9 , 575.7)
62	524.4 (405.6 , 667.2)	507.8 (392.7 , 646.0)
63	700.2 (564.5 , 858.8)	495.8 (384.2 , 629.6)
64	607.4 (487.2 , 748.4)	589.0 (472.4 , 725.7)
65	670.7 (537.9 , 826.3)	528.1 (413.2 , 665.1)
66	759.3 (601.2 , 946.3)	599.5 (462.7 , 764.1)
67	902.3 (735.7 , 1095.3)	619.1 (485.3 , 778.4)
68	792.5 (627.4 , 987.7)	623.2 (482.9 , 791.4)
69	908.6 (725.8 , 1123.5)	631.2 (486.1 , 806.1)
70	841.5 (653.5 , 1066.8)	751.1 (579.7 , 957.3)
71	864.9 (671.6 , 1096.4)	675.1 (513.9 , 870.9)
72	1216.9 (985.7 , 1486.0)	706.5 (544.1 , 902.2)

73	921.3 (719.6 , 1162.2)	760.5 (588.2 , 967.6)
74	993.6 (778.8 , 1249.3)	629.8 (471.8 , 823.8)
75	994.0 (773.4 , 1258.0)	914.7 (718.3 , 1148.4)
76	744.2 (548.8 , 986.8)	782.2 (599.7 , 1002.8)
77	898.9 (679.0 , 1167.3)	675.1 (502.6 , 887.6)
78	1001.4 (756.5 , 1300.4)	580.1 (414.4 , 789.9)
79	1054.0 (800.3 , 1362.5)	663.9 (487.8 , 882.9)
80	955.1 (704.2 , 1266.3)	546.7 (384.9 , 753.5)
81	1121.4 (840.0 , 1466.8)	725.1 (534.6 , 961.3)
82	866.1 (609.8 , 1193.8)	600.8 (423.0 , 828.1)
83	1469.9 (1104.3 , 1918.0)	521.5 (351.9 , 744.5)
84	1144.5 (805.8 , 1577.6)	456.2 (292.3 , 678.8)
85	1221.7 (860.2 , 1684.0)	561.1 (369.7 , 816.3)
86	947.4 (613.1 , 1398.6)	537.3 (344.3 , 799.5)
87	1002.0 (635.2 , 1503.6)	544.7 (345.3 , 817.4)
88	670.5 (357.0 , 1146.5)	507.9 (305.8 , 793.2)
89	941.2 (526.8 , 1552.3)	318.4 (158.9 , 569.6)
90	635.5 (290.6 , 1206.4)	471.6 (264.0 , 777.9)
91	790.8 (379.2 , 1454.4)	330.6 (158.5 , 607.9)
92	937.7 (377.0 , 1931.9)	333.5 (122.4 , 726.0)
93	1468.9 (590.6 , 3026.6)	235.2 (48.5 , 687.4)
94	541.0 (65.5 , 1954.4)	0
95	638.4 (77.3 , 2306.1)	109.0 (2.8 , 607.3)
96	422.3 (10.7 , 2352.7)	264.8 (32.1 , 956.6)
97	0	515.5 (106.3 , 1506.4)
98	0	476.2 (57.7 , 1720.1)
99	1325.1 (33.5 , 7382.7)	680.9 (82.5 , 2459.5)
100	0	0

Incidence of COPD in Lothian COPD Cohort database for Scottish patients:

Table S6

Incidence of COPD in Scotland derived from Lothian COPD Cohort database. As the denominators were very small for each year group, data was averaged over 5 year age groups, and over the ages 85-100.

Incidence rate of COPD in the general population in Scotland 2009		
Age	Male	Female
35-39	0.000	0.000
40-44	0.001	0.001
45-49	0.001	0.001
50-54	0.002	0.002
55-59	0.004	0.004
60-64	0.006	0.005
65-69	0.009	0.007
70-74	0.010	0.008
75-79	0.011	0.009
80-84	0.011	0.007
85+	0.008	0.004

Prevalence of COPD in CPRD database for English patients:

The study population for the point prevalence calculation was the same as for the incidence. calculation.

Numerator= all patients in the denominator population who have a first record of COPD at any time in their patient record prior to 01/01/2011.

Denominator=all patients in the study population

Point prevalence calculation was performed dividing the number of patients in the numerator by the number of patients in the denominator. Prevalence data is presented in S7 per 100,000 patients with 95% CI calculated using the Poisson distribution. Results are stratified by gender and age.

Table S7

Prevalence of COPD in England from Clinical Practice Research Datalink 2011.

Prevalence rate per 100,000 cases (95% CI)		
Age	Males	Females
35	92.0 (47.6 , 160.7)	152.7 (93.3 , 235.8)
36	124.7 (72.7 , 199.7)	66.9 (30.6 , 126.9)
37	139.4 (85.2 , 215.3)	163.9 (103.9 , 245.9)
38	246.3 (173.4 , 339.4)	183.2 (120.8 , 266.6)
39	244.2 (173.7 , 333.8)	160.7 (104.0 , 237.2)
40	190.9 (130.6 , 269.5)	236.4 (168.1 , 323.2)
41	239.9 (171.4 , 326.7)	361.6 (275.3 , 466.4)
42	312.6 (234.2 , 408.9)	345.9 (262.0 , 448.1)
43	457.9 (362.5 , 570.6)	298.2 (221.3 , 393.1)
44	396.3 (308.4 , 501.6)	488.3 (389.5 , 604.5)
45	412.1 (322.4 , 518.9)	428.7 (337.2 , 537.4)
46	484.1 (387.7 , 597.1)	599.8 (490.6 , 726.1)
47	624.2 (514.4 , 750.4)	618.2 (507.6 , 745.8)
48	597.9 (489.1 , 723.9)	731.5 (609.4 , 870.9)
49	849.7 (717.9 , 998.7)	969.1 (825.6 , 1130.3)
50	787.4 (658.8 , 933.8)	1061.6 (909.3 , 1232.1)
51	985.8 (839.8 , 1149.8)	1075.2 (920.9 , 1247.9)
52	1206.2 (1041.2 , 1389.9)	1408.9 (1227.7 , 1609.4)
53	1502.0 (1316.1 , 1706.8)	1393.4 (1212.6 , 1593.7)
54	1470.1 (1283.9 , 1675.8)	1669.6 (1468.2 , 1890.8)
55	1689.8 (1483.2 , 1917.2)	1751.2 (1541.7 , 1981.3)
56	2019.8 (1791.3 , 2269.4)	2366.7 (2116.4 , 2638.5)
57	2278.0 (2033.7 , 2543.5)	2396.8 (2145.9 , 2669.0)
58	2451.5 (2197.8 , 2726.4)	2681.2 (2415.3 , 2968.4)
59	3040.2 (2756.0 , 3345.7)	2760.1 (2487.5 , 3054.6)
60	2959.3 (2674.3 , 3266.4)	3418.8 (3112.2 , 3747.5)
61	3702.1 (3384.0 , 4041.9)	3472.9 (3168.2 , 3799.0)
62	3947.1 (3622.0 , 4293.5)	3702.7 (3392.1 , 4034.0)
63	4109.5 (3785.3 , 4454.0)	3858.7 (3547.8 , 4189.5)
64	4732.4 (4400.8 , 5082.3)	4274.2 (3963.2 , 4603.1)

65	5121.9 (4760.9 , 5503.0)	4547.6 (4211.9 , 4902.9)
66	6037.8 (5600.6 , 6500.1)	4925.2 (4535.4 , 5339.5)
67	6286.9 (5858.5 , 6738.4)	5673.2 (5272.3 , 6096.5)
68	7180.7 (6695.8 , 7691.4)	5646.0 (5227.4 , 6089.1)
69	7589.7 (7075.3 , 8131.6)	5839.5 (5401.4 , 6303.6)
70	7473.9 (6925.4 , 8054.3)	6810.5 (6301.9 , 7349.2)
71	8378.5 (7792.2 , 8997.3)	6519.3 (6022.8 , 7045.8)
72	8704.3 (8108.5 , 9332.2)	7241.5 (6728.8 , 7783.0)
73	9778.5 (9144.1 , 10445.4)	7168.4 (6647.5 , 7719.3)
74	9398.4 (8759.5 , 10071.6)	7579.6 (7037.4 , 8152.4)
75	9158.3 (8510.7 , 9842.2)	7440.4 (6893.2 , 8019.6)
76	10352.1 (9640.5 , 11102.3)	7706.0 (7143.2 , 8301.3)
77	10050.6 (9337.0 , 10804.2)	8147.5 (7557.1 , 8771.7)
78	10517.6 (9751.2 , 11328.2)	7821.2 (7215.7 , 8464.0)
79	10317.8 (9552.4 , 11128.3)	7706.6 (7113.7 , 8335.7)
80	10994.9 (10173.1 , 11865.3)	8286.5 (7661.2 , 8949.3)
81	11872.4 (10997.3 , 12798.6)	8541.3 (7900.6 , 9220.2)
82	11408.1 (10505.3 , 12367.8)	8156.7 (7509.0 , 8845.3)
83	12755.2 (11739.0 , 13835.9)	8489.7 (7807.0 , 9216.2)
84	12132.6 (11074.6 , 13264.5)	7477.9 (6807.2 , 8196.8)
85	11844.6 (10769.5 , 12997.9)	8149.2 (7422.2 , 8928.2)
86	12018.2 (10863.5 , 13262.2)	7854.0 (7113.0 , 8651.3)
87	11655.6 (10437.2 , 12977.2)	7025.4 (6304.5 , 7806.0)
88	11767.1 (10445.0 , 13210.3)	7120.4 (6358.1 , 7949.0)
89	11255.6 (9832.9 , 12826.4)	6604.5 (5838.7 , 7442.7)
90	11086.4 (9599.5 , 12738.3)	6471.2 (5686.2 , 7334.3)
91	10639.6 (9113.2 , 12348.6)	6857.9 (6035.7 , 7760.9)
92	9776.8 (7881.5 , 11990.4)	6544.6 (5515.9 , 7709.5)
93	8373.6 (6253.8 , 10980.9)	6392.4 (5206.7 , 7767.4)
94	8805.0 (6345.9 , 11901.8)	5705.5 (4480.0 , 7162.7)
95	8148.1 (5608.8 , 11443.0)	4148.3 (3048.0 , 5516.3)
96	7142.9 (4476.4 , 10814.4)	3415.2 (2336.0 , 4821.2)
97	5357.1 (2768.1 , 9357.9)	5020.9 (3516.6 , 6951.1)
98	4929.6 (1981.9 , 10156.8)	4212.5 (2670.3 , 6320.8)
99	8000.0 (3453.8 , 15763.2)	4134.4 (2363.1 , 6714.0)
100	13114.8 (5662.0 , 25841.3)	6227.1 (3627.5 , 9970.2)

Prevalence of COPD in Practice Team Information Database for Scottish patients:

Table S8

Diagnosis of COPD in Scotland, in the Practice Team Information dataset was based on one or more of the following Read Codes

Read code	Read code description
H3...	Chronic obstructive pulmonary disease
H31..	Chronic bronchitis
H310.	Simple chronic bronchitis
H3100	Chronic catarrhal bronchitis
H310z	Simple chronic bronchitis NOS
H311.	Mucopurulent chronic bronchitis
H3110	Purulent chronic bronchitis
H3111	Fetid chronic bronchitis
H311z	Mucopurulent chronic bronchitis NOS
H312.	Obstructive chronic bronchitis
H3120	Chronic asthmatic bronchitis
H3121	Emphysematous bronchitis
H3123	Bronchiolitis obliterans
H312z	Obstructive chronic bronchitis NOS
H313.	Mixed simple and mucopurulent chronic bronchitis
H31y.	Other chronic bronchitis
H31y1	Chronic tracheobronchitis
H31yz	Other chronic bronchitis NOS
H31z.	Chronic bronchitis NOS
H32..	Emphysema
H320.	Chronic bullous emphysema
H3200	Segmental bullous emphysema
H3201	Zonal bullous emphysema
H3202	Giant bullous emphysema
H3203	Bullous emphysema with collapse
H320z	Chronic bullous emphysema NOS
H321.	Panlobular emphysema
H322.	Centrilobular emphysema
H32y.	Other emphysema
H32y0	Acute vesicular emphysema
H32y1	Atrophic (senile) emphysema
H32y2	MacLeod's unilateral emphysema
H32yz	Other emphysema NOS
H32z.	Emphysema NOS
H36..	Mild chronic obstructive pulmonary disease
H37..	Moderate chronic obstructive pulmonary disease
H38..	Severe chronic obstructive pulmonary disease
H39..	Very severe chronic obstructive pulmonary disease
H3A..	End stage chronic obstructive airways disease
H3y..	Other specified chronic obstructive airways disease
H3z..	Chronic obstructive airways disease NOS

Table S9

Prevalence of COPD in Scotland from Practice Team Information 2011.

COPD prevalence rate as fraction of general population for Scotland		
Age	male	Female
0-4	0.002	0.002
5-9	0.000	0.001
10-14	0.001	0.000
15-19	0.000	0.001
20-24	0.000	0.000
25-29	0.001	0.001
30-34	0.001	0.002
35-39	0.002	0.002
40-44	0.003	0.004
45-49	0.008	0.010
50-54	0.016	0.021
55-59	0.028	0.030
60-64	0.049	0.053
65-69	0.057	0.070
70-74	0.094	0.090
75-79	0.097	0.084
80-84	0.094	0.080
85+	0.073	0.046

Smoking data

Smoking prevalence in England

Table S10

Smoking prevalence in England from Health Survey England 2011.

Age	Males			Females		
	Never Smoker	Current smoker	Former Smoker	Never Smoker	Current smoker	Former Smoker
0-4	1	0	0	1	0	0
5-9	1	0	0	1	0	0
10-14	0.974	0.017	0.009	0.974	0.017	0.009
15-19	0.8	0.18	0.02	0.77	0.19	0.04
20-24	0.6	0.3	0.1	0.65	0.28	0.07
25-29	0.59	0.26	0.15	0.61	0.21	0.18
30-34	0.59	0.26	0.15	0.61	0.21	0.18
35-39	0.53	0.25	0.22	0.55	0.23	0.22
40-44	0.53	0.25	0.22	0.55	0.23	0.22
45-49	0.53	0.25	0.22	0.55	0.23	0.22
50-54	0.53	0.2	0.27	0.58	0.18	0.24
55-59	0.53	0.2	0.27	0.58	0.18	0.24
60-64	0.41	0.14	0.45	0.58	0.12	0.3
65-69	0.41	0.14	0.45	0.58	0.12	0.3
70-74	0.41	0.14	0.45	0.58	0.12	0.3
75-79	0.41	0.14	0.45	0.58	0.12	0.3
80-84	0.41	0.14	0.45	0.58	0.12	0.3
85+	0.41	0.14	0.45	0.58	0.12	0.3

Smoking prevalence in Scotland

Table S11

Smoking prevalence in Scotland from Scottish Health Survey 2011.

Scottish Health Survey 2011						
Age	Males			Females		
	Never Smoker	Current smoker	Former Smoker	Never Smoker	Current smoker	Former Smoker
0-4	1	0	0	1	0	0
5-9	1	0	0	1	0	0
10-14	0.9	0.03	0.07	0.79	0.03	0.07
15-19	0.8	0.19	0.02	0.81	0.15	0.04
20-24	0.65	0.32	0.03	0.57	0.35	0.08
25-29	0.55	0.37	0.07	0.63	0.26	0.1
30-34	0.53	0.31	0.16	0.59	0.24	0.17
35-39	0.55	0.31	0.14	0.56	0.28	0.16
40-44	0.52	0.26	0.21	0.6	0.22	0.18
45-49	0.54	0.23	0.23	0.6	0.25	0.15
50-54	0.45	0.27	0.28	0.54	0.25	0.21
55-59	0.52	0.19	0.29	0.46	0.3	0.25
60-64	0.46	0.25	0.28	0.48	0.24	0.28
65-69	0.35	0.17	0.48	0.5	0.18	0.31
70-74	0.43	0.12	0.45	0.55	0.11	0.34
75-79	0.41	0.07	0.52	0.56	0.09	0.35
80-84	0.39	0.11	0.5	0.62	0.07	0.31
85+	0.49	0.07	0.45	0.67	0.06	0.27

Table S12

Proportion of the total population who were long term smokers who had successfully stopped smoking for more than a year, from the Smoking Toolkit Study November 2006 to February 2013. These are unweighted data.

Stop smoking probabilities		
Age	Male	Female
16	0.008838	0.012678
17	0.012632	0.015385
18	0.01586	0.015625
19	0.024052	0.027136
20	0.032772	0.034547
21	0.034138	0.035626
22	0.039883	0.048387
23	0.025472	0.048159
24	0.042268	0.051064
25	0.054808	0.063479
26	0.049485	0.066079
27	0.058937	0.074614
28	0.072939	0.060536
29	0.089165	0.081633
30	0.071261	0.07437
31	0.109215	0.083265
32	0.087437	0.088785
33	0.077519	0.087248
34	0.09147	0.087665
35	0.090211	0.094229
36	0.092147	0.087747
37	0.091564	0.09304
38	0.094778	0.100781
39	0.086646	0.092776
40	0.09276	0.089343
41	0.107368	0.088666
42	0.13252	0.096195
43	0.110029	0.103886
44	0.112407	0.101106
45	0.09537	0.090037
46	0.124248	0.090196
47	0.110683	0.099915
48	0.10582	0.119863
49	0.123302	0.105455
50	0.12087	0.088531
51	0.131673	0.099198
52	0.14524	0.132576

53	0.122334	0.110436
54	0.148536	0.118024
55	0.137891	0.132753
56	0.177966	0.123223
57	0.161446	0.14949
58	0.190255	0.146961
59	0.185227	0.147835
60	0.185083	0.140845
61	0.205567	0.16479
62	0.213779	0.168856
63	0.243902	0.18738
64	0.236923	0.20339
65	0.244094	0.177509
66	0.296175	0.164294
67	0.276201	0.170166
68	0.24847	0.19697
69	0.277167	0.196742
70	0.245902	0.168591
71	0.296978	0.185526
72	0.288708	0.157418
73	0.306494	0.160602
74	0.265252	0.167082
75	0.279188	0.170792
76	0.280654	0.177746
77	0.31811	0.181679
78	0.281205	0.159193
79	0.309524	0.163235
80	0.27069	0.179104
81	0.292517	0.182773
82	0.369727	0.180077
83	0.322997	0.189542
84	0.37987	0.183673
85	0.308511	0.202985
86	0.322449	0.162866
87	0.31383	0.180851
88	0.275168	0.156098
89	0.346457	0.155172
90	0.316327	0.234375
91	0.528302	0.220779
92	0.380952	0.196721
93	0.333333	0.233333
94	0.214286	0.190476
95	0.5	0.076923
96	0.333333	0.181818

97	0.4	0.2
98	0.6	0.111111
99	0.5	0

Maintenance costs

The aim was to find a total cost of all medications and services required by the average English or Scottish patient in each COPD severity stage. In a cost utility analysis for the drug indacaterol the cost data per item was published.[1] Alongside were the average annual usage statistics per severity level of COPD that came either from analysis of a large COPD patient database or from expert opinion via a Delphi-like process. Cost multiplied by usage per average patient of each severity level gives a total maintenance cost per severity level. The costs came from the 2011 edition of Unit costs of health and social care from the Personal Social Services Research Unit.[2]

Table S13

Item	Cost £	Mild use	Cost mild £	Mod use	Cost mod £	Severe use	Cost severe £	Very severe use	Cost very severe £
flu vaccine	14.2	0.73	10.366	0.73	10.366	0.73	10.366	0.73	10.366
pneumovacc	46.75	0.69	32.2575	0.69	32.2575	0.69	32.2575	0.69	32.2575
Theophylline (no. of scripts)	3.43	0.26	0.8918	0.32	1.0976	0.73	2.5039	1.63	5.5909
Mucolytics	21.85	0.35	7.6475	0.4	8.74	0.8	17.48	2.05	44.7925
oral corticosteroids	8.79	0.88	7.7352	0.96	8.4384	1.7	14.943	2.7	23.733
short acting beta agonists	5.98	3.74	22.3652	4.65	27.807	6.87	41.0826	9.78	58.4844
Inhaled corticosteroids	12.12	0.89	10.7868	0.81	9.8172	0.71	8.6052	0.62	7.5144
short acting antimuscarinics	10.24	0.59	6.0416	0.65	6.656	0.91	9.3184	1.19	12.1856
leukotriene receptor antagonists	31.77	0	0	0	0	0.37	11.7549	0	0
Pulmonary rehabilitation	1017.27	0.02	20.3454	0.03	30.5181	0.06	61.0362	0.09	91.5543
GP visits	36	15.05	541.8	15.76	567.36	16.2	583.2	16.16	581.76
Outpatient respiratory specialist visit	134.61	0	0	0	0	2	269.22	4	538.44
Spirometry	51.38	1	51.38	2	102.76	2	102.76	4	205.52
Total			711.617		805.8178		1164.528		1612.199

Relative risk values of death from COPD by age and sex

Relative risk values of death from COPD by age and sex were generated using CPRD and a time-to-event analysis with Cox regression. These values were then used with the total mortality and prevalence to calculate country, age and sex specific excess mortality rates.

In the 2005 Dutch Model, in order to calculate this excess mortality, data from the original GPRD (UK data) were used with Poisson regression to model age and sex related relative-risks for COPD. These were then substituted into the formulae with the Dutch total mortality from National Registers to obtain Dutch excess mortality.

A similar calculation had been undertaken in 2007 to obtain UK COPD excess mortality for the DYNAMO-HIA project.[3] These excess mortality figures were obtained to be used as a sensitivity analysis.

However, it was decided to generate updated estimates of the relative risks of death from COPD by age and sex using the available CPRD database. This was because the original relative risks of death in the original model were over 10 years old and it was believed that an update would more closely reflect the current risk of death from COPD given current treatments. A time to event analysis was undertaken using Cox-regression to generate these relative risks then the excess mortality by age and sex was calculated by substitution again into the formulae which are shown below with the prevalence data from CPRD (England) and PTI (Scotland), respectively.

The CPRD database was derived from CPRD data and included cases and age and sex matched controls as per Kotz et al[4]. COPD patients had been identified using relevant Read Codes as above. Then this file had been linked by a unique patient identifier number to a mortality file which included the date of death. The person-time each individual contributed to the cohort was calculated and an analysis conducted from time of inclusion to time of either event or censoring (when data ceased to be submitted to CPRD for that specific practice).

The results for the coefficients in the modelling equation are shown in Table S14. The parameters were age (AGEA), sex (GENDERA) and a marker as to whether the patient had COPD or not and interaction terms (AGEGENDER, AGECOPD, GENDERCOPD, GENDERCOPDAGE). The outcome was death over the total time for which data were available. The coefficient with the most influence on whether or not a patient died was whether the patient had a diagnosis of COPD (odds ratio 9.4).

Table S14

Parameters for Cox Regression Model of COPD patient survival

	B	SE	Wald	Df	Sig.	Exp(B)
GENDERA	-.894	.228	15.352	1	.000	.409
AGEA	.054	.002	835.708	1	.000	1.055
AGEGENDER	.008	.003	8.946	1	.003	1.008
AGECOPD	-.018	.002	62.812	1	.000	.982
GENDERCOPD	.458	.277	2.739	1	.098	1.581
GENDERCOPDAGE	-.005	.003	2.231	1	.135	.995
Marker	2.245	.185	146.713	1	.000	9.441

These coefficients were then used to calculate relative risks for patients in the middle of each five year age bracket by multiplying out the model for every x. Where x was a patient of a specific age and gender (gender=1 for females and gender =0 for males) and COPD marker (1=COPD, 0=no COPD).

Hazard at age and sex x= $e^{(\text{BMarkerx * BGENDERAx * BAGEAx *BAGEGENDERx * BAGECOPDx * BGENDERCOPDx * BGENDERCOPDAGE})}$

Relative risk was calculated by finding the risk (or hazard) at age and sex x with COPD then dividing this by the risk at age and sex x without COPD.

Table S15

Age specific relative risk of dying per year if patient has COPD compared to an age and sex matched control as obtained from CPRD data extract

	Male	female
Age	relative risk	relative risk
40-44	4.39	5.62
45-49	4.01	5.01
50-54	3.67	4.46
55-59	3.35	3.98
60-64	3.06	3.54
65-69	2.80	3.16
70-74	2.56	2.82
75-79	2.34	2.51
80-84	2.14	2.24
85+	1.95	1.99

Excess Mortality

The Dutch COPD Model calculated country, age and sex specific COPD excess mortality[5] as follows.

<u>Definitions:</u>

M_1= All cause mortality of COPD patients

M_0= All cause mortality in those without COPD

R= relative risk of death from COPD

p=Prevalence

$R=M_1/M_0$ therefore $M_1 = M_0R$

Total population mortality= mortality in those with COPD + mortality without COPD

$= M_1p+M_0(1-p)$

Substituting for M_1 $=M_0Rp+M_0(1-p)$

Factorising for M_0 $=M_0\{Rp+(1-p)\}$

Rearranging M_0=total population mortality/$\{Rp+(1-p)\}$

Then "Excess mortality" $=(R-1)*M_0$ where M_0 is defined as above.

Table S16

Excess mortality for COPD in England

Excess Mortality England		
Age	males	females
40	0.007429	0.0058
41	0.007417	0.005767
42	0.007399	0.005771
43	0.007363	0.005784
44	0.007378	0.005734
45	0.009605	0.007855
46	0.009584	0.007803
47	0.009544	0.007797
48	0.009552	0.007763
49	0.009481	0.007692
50	0.013675	0.011019
51	0.013604	0.011014
52	0.013526	0.010893
53	0.013424	0.010898
54	0.013435	0.0108
55	0.019774	0.01507
56	0.019627	0.014812
57	0.019514	0.0148
58	0.019439	0.014684
59	0.019187	0.014652
60	0.027095	0.020077
61	0.026709	0.020052
62	0.026584	0.019945
63	0.026502	0.019873
64	0.026192	0.019683
65	0.038491	0.028184
66	0.037918	0.027976
67	0.037765	0.027573
68	0.037227	0.027588
69	0.036986	0.027485
70	0.058225	0.040887
71	0.057498	0.04108
72	0.057241	0.040604
73	0.056409	0.040652
74	0.0567	0.040385
75	0.086707	0.06436
76	0.08549	0.064129
77	0.085794	0.063748
78	0.085323	0.064029
79	0.085524	0.064129

80	0.14146	0.108409
81	0.140215	0.108099
82	0.140871	0.108567
83	0.138985	0.108162
84	0.13985	0.109402
85+	0.230752	0.182546

Table S17

Excess mortality for COPD in Scotland

Excess Mortality Scotland		
Age	males	Females
40	0.012096	0.007387
41	0.012096	0.007387
42	0.012096	0.007387
43	0.012096	0.007387
44	0.012096	0.007387
45	0.012945	0.01076
46	0.012945	0.01076
47	0.012945	0.01076
48	0.012945	0.01076
49	0.012945	0.01076
50	0.016582	0.014891
51	0.016582	0.014891
52	0.016582	0.014891
53	0.016582	0.014891
54	0.016582	0.014891
55	0.02474	0.018765
56	0.02474	0.018765
57	0.02474	0.018765
58	0.02474	0.018765
59	0.02474	0.018765
60	0.033493	0.024767
61	0.033493	0.024767
62	0.033493	0.024767
63	0.033493	0.024767
64	0.033493	0.024767
65	0.050679	0.035558
66	0.050679	0.035558
67	0.050679	0.035558
68	0.050679	0.035558
69	0.050679	0.035558
70	0.074428	0.051242
71	0.074428	0.051242
72	0.074428	0.051242
73	0.074428	0.051242
74	0.074428	0.051242
75	0.10509	0.080003
76	0.10509	0.080003
77	0.10509	0.080003
78	0.10509	0.080003
79	0.10509	0.080003

80	0.175954	0.128891
81	0.175954	0.128891
82	0.175954	0.128891
83	0.175954	0.128891
84	0.175954	0.128891
85+	0.274239	0.21434

The relative risks (Table S14) were combined with the total mortality (all-cause mortality) from the Office of National Statistics England for 2011 and the General Registrar of Scotland for 2011 according to the above formulae and each 1 year age and sex interval in order to generate the excess mortality. The modelled excess mortality with these updated relative risks for England and Scotland were used to run the model base cases (i.e. run of the model without sensitivity analysis). Then the UK modelled excess mortality as used in the DYNAMO-HIA study was used as a sensitivity analysis.

Additional probabilistic sensitivity analysis

Probabilistic sensitivity analysis was undertaken to estimate the effects of uncertainty around the new input parameters on the outcomes. Monte Carlo simulation was conducted by drawing random values from the distributions for incidence and prevalence, after which the model was run for each set of parameters and results collected. The current analyses were based on 1000 simulations, providing the 95% uncertainty interval around the prevalence, costs and number of deaths.

Aim

The aim was to generate age-specified incidence and prevalence probability values for England and Scotland taking account of uncertainty.

Calculation

A smooth curve was calculated through the age-specific data using R-routine smooth.spline (spline-based method R Version 3.0.1) The smoothing parameter used was spar0, and the value was selected to optimize both fit and smoothness. The same procedure was then applied for each generated random curve. Prevalence parameters were drawn from binomial distributions for each age-class with parameters sample size and observed prevalence probability numbers. Incidence parameter was drawn from Poisson distributions with parameter observed event number. Then a smooth curve was calculated through the randomly drawn age-specific data using smoothing parameters: Scotland spar0=0.25, England spar0=0.6. The model was then run 1000 times with these probabilistic inputs to give 1000 outputs and mean result and 95% uncertainty intervals were identified.

References

1 Price, D. *et al.* A UK-based costs utility analysis of Indacaterol, A Once Daily Maintenance Bronchodilator for Patients with COPD, using real world evidence on resource use. *Applied Health Economics Helath Policy* **11**, 259-274 (2013).

2 Curtis, L. Unit costs of health and social care. (Personal Social Services Research Unit, University of Kent, 2011).

3 Lhachimi, S. *et al.* DYNAMO-HIA--a Dynamic Modeling tool for generic Health Impact Assessments. *PLoS One* **7**, e33317. doi: 33310.31371/journal.pone.0033317. Epub 0032012 May 0033310. (2012).

4 Kotz, D., Simpson, C. R., Viechtbauer, W., van Schayck, O. C. & Sheikh, A. Development and validation of a model to predict the 10-year risk of general practitioner-recorded COPD. *NPJ Prim Care Respir Med* **24**, 14011, doi:10.1038/npjpcrm.2014.11 (2014).

5 Barendregt, J. J., Baan, C. A. & Bonneux, L. An indirect estimate of the incidence of non-insulin-dependent diabetes mellitus. *Epidemiology* **11**, 274-279 (2000).
